# Energy Aware Cluster-Based Routing in Flying Ad-Hoc Networks

**DOI:** 10.3390/s18051413

**Published:** 2018-05-03

**Authors:** Farhan Aadil, Ali Raza, Muhammad Fahad Khan, Muazzam Maqsood, Irfan Mehmood, Seungmin Rho

**Affiliations:** 1 Department of Computer Science, COMSATS Institute of Information Technology, Attock 43600, Pakistan; farhan.aadil@ciit-attock.edu.pk (F.A.); fa16-rcs-019@ciit-attock.edu.pk (A.R.); m.fahad@ciit-attock.edu.pk (M.F.K.); Muazzam.maqsood@ciit-attock.edu.pk (M.M.); 2 Department of Software, Sejong University, Seoul 143-747, Korea; 3 Department of Media Software, Sungkyul University, Anyang 430-742, Korea; smrho@sungkyul.ac.kr

**Keywords:** FANET, routing, clustering, transmission range optimization, energy optimization

## Abstract

Flying ad-hoc networks (FANETs) are a very vibrant research area nowadays. They have many military and civil applications. Limited battery energy and the high mobility of micro unmanned aerial vehicles (UAVs) represent their two main problems, i.e., short flight time and inefficient routing. In this paper, we try to address both of these problems by means of efficient clustering. First, we adjust the transmission power of the UAVs by anticipating their operational requirements. Optimal transmission range will have minimum packet loss ratio (PLR) and better link quality, which ultimately save the energy consumed during communication. Second, we use a variant of the K-Means Density clustering algorithm for selection of cluster heads. Optimal cluster heads enhance the cluster lifetime and reduce the routing overhead. The proposed model outperforms the state of the art artificial intelligence techniques such as Ant Colony Optimization-based clustering algorithm and Grey Wolf Optimization-based clustering algorithm. The performance of the proposed algorithm is evaluated in term of number of clusters, cluster building time, cluster lifetime and energy consumption.

## 1. Introduction

A FANET is a derived form of vehicular ad-hoc network (VANET) and mobile ad-hoc network (MANET). In FANETs, UAVs are network nodes. FANETs follow the same peer to peer communication. Apart from many common characteristics, there are also some differences between FANETs, MANETs and VANETs. FANETs have fast mobility, rapid topological changes, 3D environmental conditions, different mobility patterns and terrain structure [1]. The highly dynamic topology and the harsh FANET environment produce many challenges for networking and communication [2]. As the nodes fly in the air, they have clear line-of-sight and a relatively large distance from each other.

Because of design limitations, UAVs are equipped with a small battery. This small battery can sustain a flight time of about 30 min [3,4]. Apart from the limited battery energy, UAVs have other limited resources such as computational power and channel bandwidth. These scarce resources hinder the wide application of UAVs. These resources can be utilized efficiently by devising a communication mechanism among UAVs such that it has minimum routing overhead, maximum throughput and low computational complexity [5].

Several techniques have been used for communication in an ad-hoc network. These techniques can be categorized into proactive routing, reactive routing and cluster-based routing. In proactive routing, routing tables are maintained which contain routes to the other nodes in the network. Routing tables are updated through the exchange of periodic messages. On the other hand, reactive routing protocols don’t maintain the routing table. Whenever a node requires sending data, it finds a new route. Upon the disruption of the current route, it finds a new route again. In the highly dynamic environments where nodes change their position very quickly and frequently, maintaining the latest routing table or finding a new route, again and again, represents a huge communication overhead. This communication overhead reduces network throughput, introduces additional delays and wastes the limited battery energy of UAVs [3].

One remedy for this scarce resources problem is clustering. Clustering is an approach for arranging nodes having same geographical neighborhood, into multiple groups. It helps to make the network more scalable, reduce routing overhead and maximize the throughput [6]. This low routing overhead will save the UAVs’ energy as well. Although clustering has its own overhead, such as cluster formation and maintenance, this overhead is much less as compared to the other two.

In clustering, the whole network is divided into multiple logical groups or clusters. Each cluster has a leader or cluster head (CH), which is responsible for inter-cluster and intra-cluster communication. For the purpose of routing, CH acts as a first hop node and it is responsible for delivering the message to the ultimate destination.

Regardless of the potential benefits of node clustering in FANETs, the cluster formation and maintenance demand explicit exchange of control messages [6,7]. A portion of the computational network radio resources is used for cluster formation and maintenance. In the cluster formation phase, nodes exchange some local information (e.g., node ID, location and residual energy, etc.). This information is exchanged through control messages. Therefore, a portion of the network radio resources is used for cluster formation. During network clustering, nodes perform computations in order to arrange nearby located nodes into clusters and a CH is elected from each cluster. The process of cluster formation and electing CHs is very important in maintaining the cluster structure. Subsequent changes in the relative positions of cluster members (CMs) can modify the cluster structure. In order to track changes in cluster structure, CHs should always periodically broadcast their existence to its CMs, and each CM should reply back its status to the CH. This signaling for cluster maintenance also uses a part of the network radio resources.

The periodic exchange of control messages for cluster formation and maintenance is termed as cluster overhead. This overhead consumes radio resources (e.g., channel bandwidth) and wastes the UAVs’ energy. The lifetime of the cluster is a parameter, among others, that is used for evaluating the performance of a clustering model. The longer the lifetime of the cluster, the less will be the cluster maintenance overhead and more effective the model will be. The lifetime of the cluster depends upon the efficient selection of CHs.

The performance of a network using clustering depends upon its clustering algorithm. In the literature, artificial intelligence techniques have been used to divide networks into clusters. These techniques produce more optimal results, but, the main disadvantage of these techniques is that they have high computational complexity. They take too much time to converge towards an optimal solution. In the highly dynamic environment and with the limited processing power of UAVs, computationally expensive techniques are also not suitable for clustering in FANETs.

Selection of transmission power also plays a vital role in the energy consumption of UAVs. There is direct relationship between transmission power and energy consumption. Selecting a transmission power above or below an optimal value will result in more energy consumption. An optimal transmission power must be high enough to maintain good connectivity with neighbors yet low enough to avoid the wastage of energy [8].

In this paper, we propose the Energy Aware Link-based Clustering (EALC) model. EALC uses a variant of the K-Means Density [9] clustering algorithm in order to elect CHs. K-Means Density is used along with K-means for initial selection of centroids. Original K-Means Density uses only one parameter, i.e., the degree of the neighborhood, whereas EALC adds two other parameters—energy level and distance—to the neighbors for the election of an optimal CH. EALC enhances the cluster lifetime and improves the energy consumption. EALC also saves the nodes’ energy by efficiently selecting the transmission power of nodes according to the operational requirements. EALC outperforms the Ant Colony Optimization- and Grey Wolf Optimization-based clustering models in term of cluster building time, cluster lifetime and energy consumption.

The rest of the paper is organized as follows: in Section 2 a review of existing clustering models is presented, Section 3 provides a detailed description of the proposed EALC, while discussion and results are given in Section 4 and in Section 5 we conclude the paper.

## 2. Related Work

Although a FANET is a sub-domain of ad-hoc networks, however, the unique characteristics of FANETs do not allow one to use MANET and VANET clustering techniques directly. Rather new techniques are to be developed or variants of existing techniques are required which take into account the unique attributes of a UAV network. Bilal et al. [10] used the multi cluster-based approach, in which each cluster consists of a fixed number of UAVs and one UAV is elected as a CH. At the start, a node information message is exchanged among neighboring UAVs. UAVs are grouped into different clusters based on the “zone ID” field of the node information message. Each node in the cluster maintains a link quality table which contains the distance, SNR and delays to the neighbors. CH election is based on link quality information and the node with the best link quality is elected as a CH. Shi et al. proposed a novel routing protocol called Cluster-based Location Aided Dynamic Source Routing (CBLADSR) [11]. CBLADSR elects the CHs based on three parameters, i.e., relative velocity, energy level, and degree of connectivity. A cluster member having low relative speed, higher energy level and a large number of neighbor nodes will be elected as a CH. Member nodes maintain a neighbor table, listing all nodes in that particular cluster. CBLADSR uses short range and long range transmission for intra- and inter-cluster communication, respectively.

Zang and Zang proposed a mobility prediction clustering algorithm [12] for UAVs. Each node maintains a neighbor table for its one-hop neighbors. The neighbor table also contains the probability that a node will remain in its table. This probability is computed using a dictionary tree structure. Link expiration time (LET) is predicted using this probability and moment of the neighbor node. A weight is calculated for each node using the neighbor’s LET probability and degree. A node with the highest weight will be elected as a CH. Bilal et al. [13] and Rizwan et al. [14] also discussed the future direction of ad-hoc networks by using the different nature of algorithms. These suggestions are also based on the mobility pattern of nodes. Peer et al. [15] discussed the routing method by using the fuzziness in wireless multi hop networks. Nadeem et al. [16] proposed a clustering method for the selection of centroid used in the domain of recommendation system by using the different datasets.

Artificial intelligence techniques, such as Ant Colony Optimization (ACO) [17], Particle Swarm Optimization (PSO) [18] and Grey Wolf Optimization (GWO) [19], have also been used to perform clustering in ad-hoc networks. These techniques generate a population of solutions. Each solution contains the all possible nodes that can be elected as a CH. These solutions are updated iteratively based on distance from local best and global best. Adil el al. proposed an ACO-based clustering algorithm (CACONET) [20], which uses the ACO to find the optimal cluster. ACO uses the social behavior of ants in finding some foods or solution to some common problem. Vertices in the search space are the CH and each complete round gives the collection of CHs from a given environment. CACONET selects some vertices randomly and adds them into the cycle. Later it will add more vertices to the round, subject to some evaluation criteria and heuristic value. CACONET calculates the probability of selection for each node and uses the Roulette Wheel selection method to select a node to become vertices in the route. After selecting vertices, ants move over the edge and evaluate the next vertices. This process is repeated until there are no vertices left to be added to the tour. CACONET use the two objective functions: delta difference and Euclidean distance, to evaluate the fitness of each cycle. Another ACO-based algorithm called ACONET [21] has also been used for the same problem.

GWOCNETs [22,23] mimics the leadership hierarchy and hunting mechanism of grey wolves. There are four types of grey-wolves: alpha, beta, delta, and omega. An alpha wolf lies at the top of leadership hierarchy and acts as a leader of a group of wolves. All other wolves follow the alpha, beta and delta wolves to update their position. In the optimization problems, each wolf will be considered as a solution. The alpha solution will lead the other solution and will act as an optimal solution. CAVDO [24] used the feature extraction of the Dragon Fly Algorithm to solve the mentioned problem.

Although the artificial intelligence techniques produce more optimal results, the main disadvantage of these techniques is that they have high computational complexity. They take too much time to converge towards an optimal solution. They start with initial random multiple solutions and iteratively improve towards a global solution. Because of random selection and large population size, these algorithms converge very slowly. As we know that network topology changes very quickly due to the high mobility of UAVs, with the limited processing power of UAVs, these artificial intelligence techniques take too much time to produce an invalid result that can’t be used for changed network topology. Computationally expensive techniques are also not suitable for energy constrained networks.

## 3. EALC Methodology

EALC is a FANET communication model that attempts to minimize the computational and communicational overhead. The computational overhead can be reduced by keeping the clustering mechanism simpler and communicational overhead can be minimized by increasing the lifetime of the cluster. We also attempt to select optimal transmission range of UAVs according to the network requirements. Here, the network requirement means the need of minimum transmission range for a node to communicate efficiently.

**Figure 1:**
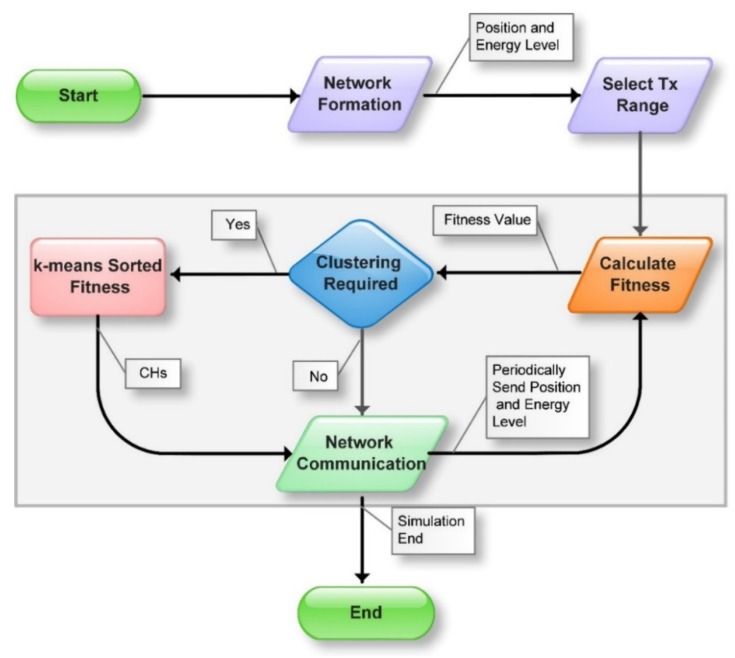
Cluster-based routing protocol for FANETs.

At the start of EALC, nodes are grouped into clusters using K-Means Sorted Fitness algorithm and communication is started through the CHs. Nodes also periodically broadcast their energy and position to CHs. During the lifecycle, if the fitness of a CH falls below some threshold (e.g., 20% below of any CM), all nodes of that cluster will be considered as unclustered nodes. If 20% of all nodes are unclustered, then clustering will be recalled. A flow chart for EALC using a variant of K-Means Density as a clustering technique is presented in Figure 1 and Algorithm 1. The key steps exhibited are explained below:
**Algorithm 1.** Cluster-based routing protocol for FANETs.STARTPosition the node in the 3D gridDivide the whole gird into 4 *sub-grids*Node *n* ∈ *N* broadcasts the *position and residual energy* information messageCalculate average *distance D* for each *n* in the same *sub-grid*Adopt optimal *P_T_* based on *D*WHILE simulation is not end    FOR each node *n* ∈ *N*      *f_val* ← Fitness (*distance, no. of neighbors, residual energy*)    END FOR    Send *f_val* to neighbors    IF uncluttered nodes > 20% of *N*      CHs ← K-meansSortedFitness (*f_val*)    ELSE      Nodes follow the *RPGM* for their movement      CMs start communication through CH    END IF    Periodically exchange *distance, no. of neighbors and*    *residual energy*END WHILEEND

### 3.1. Network Formation

The network formation starts once the UAVs start flying. Along with task-oriented sensors, UAVs are also equipped with GPS and height sensors. These two pieces of equipment provide the 3D positional information of the UAV node. We also assume that UAVs have four discrete power levels, which correspond to transmission ranges of 400, 600, 800 and 1000 m, respectively. At the start, nodes select highest power level. Later on, nodes can adopt the power level that is most suitable according to its position and the neighbor nodes. This technique is used to save node energy.

### 3.2. Selecting Transmission Range

Transmission range is the maximum acceptable distance between the transmitting node and the receiving node such that signal transmitted from the transmitting node can directly reach the receiving node with acceptable signal strength. Transmission power is the strength of the signal at the time of emission from the transmitting node. The transmission range R of a wireless node is tightly coupled with transmission power [8]. The Frist equation (Equation (1)) determines the relationship between *R* and *P_T_* [25]:

(1) $$P_{T}=P_{R}+20\log\left(\frac{4\pi R}{\lambda }\right)-G_{T}-G_{R}$$

*G_T_* and *G_R_* and represent the antenna gain of the transmitting and receiving side, respectively. *λ* represents the wavelength of the transmitted frequency and *P_R_* is the receiver sensitivity. It can be depicted by Equation (1) that the more the transmission power, the higher the range will be. Furthermore, nodes with the higher transmission range have better connectivity. On the other hand, the higher transmission power consumes more energy that ultimately reduces the lifetime of the UAV node. Higher transmission power leads to higher transmission range, but increasing the power two times does not double the transmission range. Due to the logarithmic relationship between them, increasing the power 100 times increases the range only ten times. In other words, we can say that a slight reduction in the transmission range will lead to more power savings, while setting more transmission power than the application’s requirement wastes a noteworthy amount of energy.

Somehow, if we can analyze our operational requirements and application scenario, we can find the maximum distance the nodes can get apart. Adjusting the transmission power according to the requirements, we can save a notable amount of energy that ultimately enhances the lifetime of UAV nodes.

Keeping the transmission power lower does not guarantee the optimization of the energy problem. Low power transmission provides a short communication range that results in a lower degree of the neighborhood. Even the nearby located nodes have poor connectivity and suffer from low link quality. With short transmission ranges, neighbor nodes change more rapidly. These rapid changes disturb the data route and often require the reestablishment of new routes. Establishing the new route is also an overhead in network communication. It consumes a huge amount of channel bandwidth and drains the extra energy as well. Poor link quality, changes of neighborhood and route reestablishment result in high a Packet Loss Ratio (PLR) and require re-transmission of data packets. Higher numbers of retransmissions consume even more energy because we have to send more packets to deliver the same information, so, it is desirable to keep a balance between transmission range and energy optimization. An optimal value or set of values must be calculated for each FANET application. At this optimal value, the energy consumption will be optimal. Higher or lower transmission ranges will shorten the node life.

### 3.3. SNR and PLR

Signal to noise ratio (SNR) is the ratio between the signal level and noise level. It indicates the strength of the received signal minus the noise power. Packet loss ratio (PLR) is a ratio of the total number of packets sent to the number of packets lost. In general, PLR depends on the path loss and noise interference, where path loss is determined by the transmission medium. Non-conductive materials such as buildings, hills human bodies, etc. produces absorptions and increase the path loss. The relationship between SNR, PLR and path loss can be derived as follows:

(2) $$PLR=1-{\left(1-0.5e^{-0.78125*SNR}\right)}^{8f}$$

where:

$$SNR=P_{T}-PL\left(d\right)-P_{n}$$

$$PL\left(d\right)=20\log\left(\frac{4\pi d}{\lambda }\right)$$

**Figure 2:**
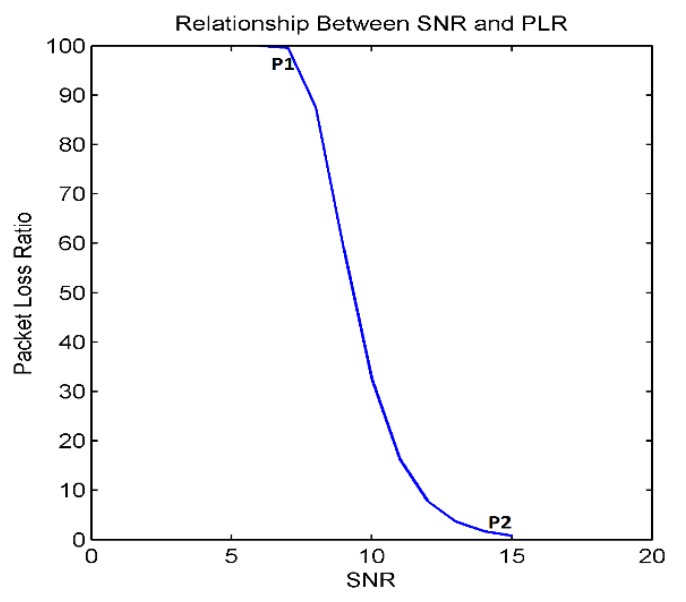
The relationship between SNR and PLR.

*P_T_* is transmission power, “*f*” is frame size, *PL*(*d*) is path loss, *P_n_* is noise power and “*d*” is the distance between transmitting and receiving nodes. Figure 2 visualizes the relationship between SNR and PLR. An increase in SNR will lower the PLR, but at points SNR > *P*2 and SNR < *P*1, there is no change in PLR. If we increase *P_T_* such that SNR > *P*2, PLR remains at a minimum. Increasing *P_T_* beyond this point does not improve PLR, but it only leads to high energy consumption. If we are able to estimate the maximum “*d*” (max transmission range) according to the application requirements, we can adjust *P_T_* such that SNR = *P*2 at maximum “*d*”. At this point, *P_T_* is low enough to achieve minimum PLR. Hence, node energy can be optimized by adjusting *P_T_* according to the operational requirements.

In order to find the transmission range requirement of the application, we divide the operation area or grid into four sub-grids. Nodes receive 3D positional information (latitude, longitude, and height) from each other. Based on this information, they calculate the average distance (using Equation (3)) from all nodes within the sub-grid:

(3) $$Avg_{Dis}=\frac{\sum_{i=1}^{n}d}{n}\;$$

“*n*” is the number of nodes in sub-grid and “*d*” is the distance of each node from all other nodes in the same sub-grid. This average distance is then used to select the transmission power level of all nodes in the sub-grid. After defining the receiver sensitivity and computing the path loss at the average distance, transmission power must be set high enough such that SNR at the outer edge of transmission range must provide minimum PLR. After computing the *P_T_* adopt the predefined power level that is the most appropriate to this *P_T_*. In this way, we select the transmission power according to an operational requirement. The selected power level is enough to maintain the contact with other nodes in the same sub-grid and low enough to save the node energy.

Nearby flying nodes are likely to maintain their neighborhood with nodes in same sub-grid for some period of time. During flying, a node may change its sub-grid and can adapt the transmission power according to the current settings of the sub-grid. The main reason for dividing the operational area into four sub-grids is that it is found to be an optimal value. If we increase the sub-grid area (i.e., decreasing the number of sub-grids), it results in long average distances which cause the setting of high transmission power. Because the nodes only communicate with or through their nearby located nodes, setting high power will waste the node energy. On the other hand, division into a greater number of sub-grids results in very small sub-grid areas, which leads to frequent movement of nodes into sub-grids and they have to adopt transmission power accordingly. Frequent switching of power levels produces fluctuation in the electric current of the UAVs’ circuitry.

### 3.4. Fitness Function

The fitness function is one of the crucial parts of an algorithm. It defines the accuracy of the algorithm. An accurate fitness function will not be biased by any parameter and provide more accurate results, which leads towards the selection of optimal CHs. As mentioned earlier, optimal CHs will enhance the cluster lifetime, which ultimately saves the network energy. After receiving the 3D position information and selecting the transmission range, each node calculates its fitness value and send it to neighbor nodes. EALC calculate fitness value using Equation (4):

(4) $$\;Fitness=\;\frac{w1\times Energy_{Res}}{\left(w2\times avg\_dis\right)\left(w3\times delta\_diff\right)}$$

*Energy_res_* is node residual energy level, “*avg_dis*” is the average distance to the neighbor nodes and *delta_diff* is the delta difference. Delta difference is used for load balancing factor. *w*1, *w*2 and *w*3 are weights for energy, average distance and delta difference respectively. Ideally, all CHs must have an equal number of nodes, but this is not possible in real world scenarios because nodes change their position very frequently which changes their degree of the neighborhood. Delta difference is the deviation of node’s degree of the neighborhood from the ideal degree. It is calculated using Equation (5):

(5) $$D_{diff}=ABS\left(Ideal_{Degree}-Node_{Degree}\right)$$

### 3.5. Weight Assignment

**Figure 3:**
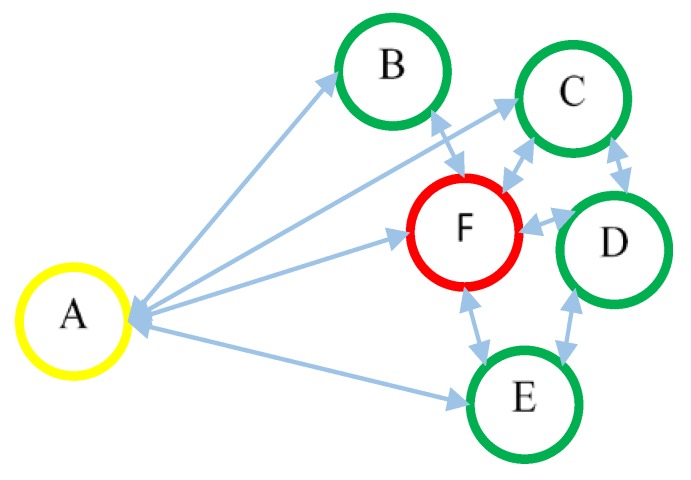
Scenarios where the static weight assignment is incorrect.

According to the best of our knowledge, all of the previous works statically assign the weight to fitness parameters based on their importance. However, this static weight assignment may bias the fitness function and provide inaccurate results. Figure 3 describes two of such scenarios in which static assignment is not suitable and biases the fitness function.

Let us suppose all the nodes are in transmission range of each other and one node is to be elected as CH based on its fitness values. We also consider the energy levels of all nodes in percentages:

*Scenario 1*: If node “A” has an energy level at 90% and all other nodes have 50%. Such high differences in energy level may cause the node “A” to be elected as a CH. It is obvious from Figure 3 that if node “A” is elected as a CH, it will consume more energy of its own as well as of its CMs because of the large distance from other nodes.

*Scenario 2*: If node “F” has the energy level of 30% and all other nodes have 50%. Because of the short distance of form other nodes, node “F” may be elected as a CH. Energy consumption rate of CH is much more than the other nodes because it has to relay the network traffic of all member nodes. If node “F” become CH, it will consume its energy more rapidly and will die out very soon or result in again calling of the clustering algorithm.

As we have seen that static assignment may lead towards the selection of inappropriate CHs. One or more parameters may bias the fitness function and provide inaccurate results in many other such scenarios. To overcome this problem, EALC dynamically assigns the weight to each parameter based on its negative impact on the fitness function. First, it normalizes each parameter value and brings it into the range from 0 to 10. Then, the deviation of each parameter from the mean value of all parameters is calculated using Equation (6). This deviation shows the negative impact of a parameter on the fitness function. Higher values of deviation point out the outlier parameter. To avoid this bias, we add some penalty during assignment of weights, based on parameter deviation from the mean value. Equation (7) computes the weight of each parameter:

(6) dev(i)=ABS(mean−parameter(i))

(7) $$w\left(i\right)=\;\frac{1}{dev\left(i\right)}$$

where “*w*” and “*dev*” are the weight and deviation of *i*th parameter respectively. Sum of all weights must be equal to “1”. By using these parameters value and their corresponding weights, the fitness value of each node is computed using fitness function (Equation (4)).

### 3.6. K-MeansSortedFitness

K-Means Sorted Fitness (a variant of K-Means Density) performs the actual clustering and elects CHs. K-Means Density is used along with K-means for initial centroids selection. It computes “k” number of optimal centroids location based on the degree of the neighborhood of data points and provides them as an input parameter for K-means. These pre-computed centroids allow the K-means to converge very quickly towards an optimal solution.

In K-Means Density, we have to provide a fixed value for “k”, whereas in FANETs, we cannot specify the “k” value, because the nodes change their position very frequently and due to this change, the network topology also changes. Each network topology requires a different number of clusters based on nodes position and transmission range. In K-Means Sorted Fitness, we consider the transmission range of CHs in order to determine the size and number of clusters.

**Figure 4:**
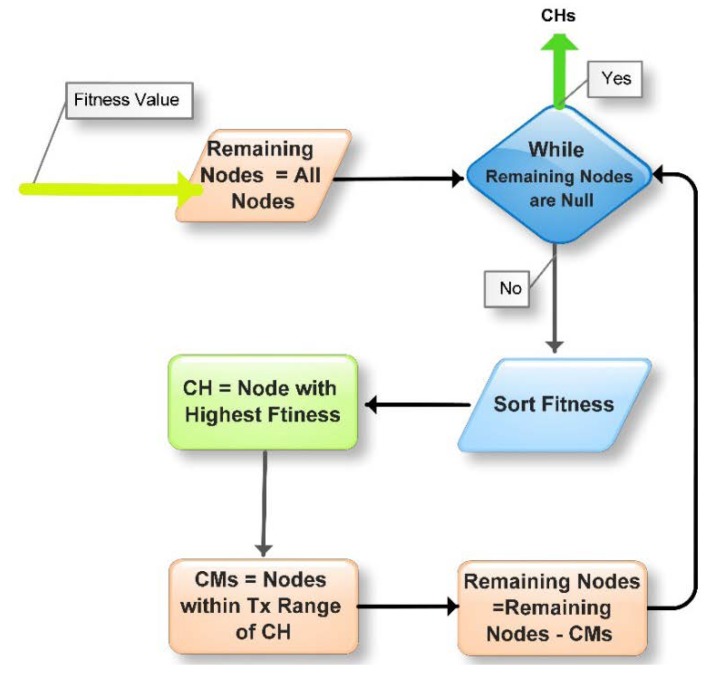
Flow chart for K-Means Sorted Fitness for clustering the network.

K-Means Sorted Fitness takes the fitness value of nodes as input and provides CHs and their associated member as output. To reduce the computational overhead of the clustering function, EALC keeps it simple as much as possible. This function takes decisions based on the fitness value. More accurate the fitness value, more optimal CHs will be elected. Figure 4 and Algorithm 2 illustrates the clustering process. First of all, the fitness value is sorted in decreasing order. A node with the highest fitness will be elected as a CH. All other nodes that are within the transmission range of the selected CH, will become its CMs. The elected and its associated member nodes will then be removed from the remaining nodes. This process of electing CHs is repeated until no remaining node is left behind. At this point in time, all nodes have adopted their role, either as a CH or a cluster member.

**Algorithm 2.** K-Means Sorted Fitness
Input: *f_val* for all *n* ∈ *N*Output: *CHs* and their affiliated *CMs*START*Remaining nodes* ← All nodes WHILE (Remaining nodes ! = 0)      Sort (*f_val)*      *CH_i_* ← node ID having highest *f_val*      *Member* ← All nodes in the transmission               range of *CH_i_*    *Remaining nodes* ← *Remaining nodes—Member*END WHILEReturns *CHs*END


### 3.7. Node Movement and Network Communication

After the clustering process, nodes start communicating and send their data to their CH. It is the responsibility of the CH to deliver the data to the intended destination, either to a node within a cluster, across the cluster or to the base station. EALC follows the reference point group mobility model RPGM [26,27]. In RPGM, all nodes follow some reference point for their movement. EALC considers the CHs as reference points and all CMs update their position according to the movement of their CHs. The process in the grey shaded area in (i.e., calculating fitness, clustering through K-Means Sorted Fitness) in Figure 1 will be repeated until the flight operation or simulation time is ended.

### 3.8. Computation Complexity of EALC

It’s a well-known fact that implementation of ACO- and GWO-based methods is always more complex as compared to EALC. They look for an optimal solution from the entire search space. However, EALC is much more suitable for the subject problem. It has very low computational complexity and easy implementation. The computational complexity of EALC for clustering the entire network can be computed as follows:

*Complexity for fitness function*: This function calculates and returns fitness for every “n” nodes. Constant “c” time is required to calculate fitness of “n” nodes:

Time for computing fitness for “n” nodes = n × c ∈ O (n)

*Complexity for k-meansSortedFitness:* This function takes fitness value of “n” nodes as input and returns CHs as output. Time complexity for the best case is when all nodes located much close to each and have direct connectivity with all nodes. At then nodes will form only a single cluster. On average, it divides the network into “k” number of clustera. In the worst case, when no node lays in the transmission range of any other node, it produces “n” number of clusters.

Body of the *While* loop in k-meansSortedFitness function can run: (4n) ∈ O (n)

Time complexity for *While* loop:

Best case O (n) × 1 ∈ O (n)
Average case O (n) × k ∈ O (kn)
Worst Case O (n) × n ∈ O (n^2^)

Total time complexity of EALC

T(EALC) = T(*fitness function) +* T(*k-meansSortedFitness*)

Best case T(EALC) = O (n) + O (n) ∈ O (n)
Average case = O (n) + O (kn) ∈ O (kn)
Worst Case = O (n) + O (n^2^) ∈ O (n^2^)

## 4. Experimental Results and Analysis

In this section, we present a performance comparison of three clustering approaches: GWO-based clustering, ACO-based clustering (CACONET) and EALC. The metrics that we used to evaluate their performance are: number of clusters, cluster building time, cluster lifetime and energy consumption. The experiments are carried out in MATLAB with the different grid-VS-node setup. Parameters settings for simulation are shown in Table 1. We conducted ten experiments with each setup and computed their average results.

### 4.1. Number of Clusters

Optimizing the energy using clustering requires the optimal number of clusters. At this optimal number of clusters, energy consumption will be optimal. If the number of clusters is less than the optimal value, more nodes will be away from CHs causing the network energy to be consumed more rapidly. If clusters are more than the optimal number of clusters, more CHs have to send data at long haul distances to communicate with the base station.

**Figure 5:**
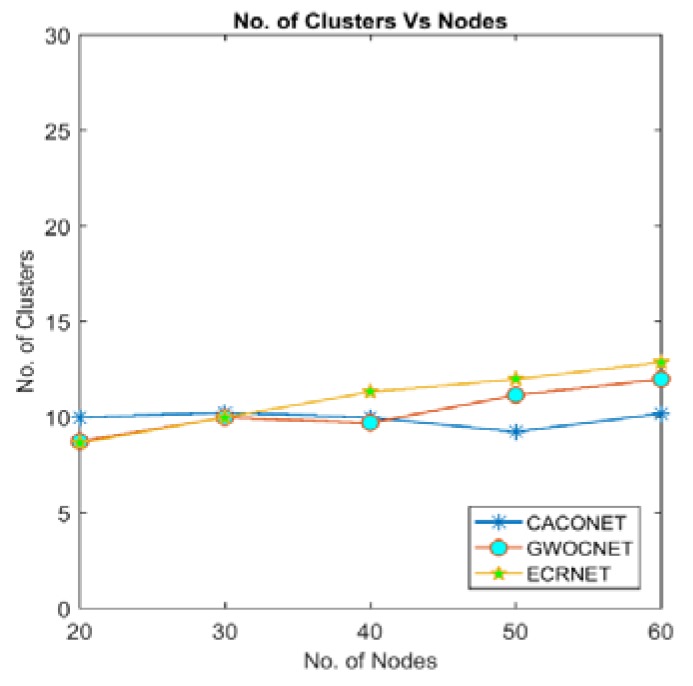
Number of clusters vs. nodes at a 2000 × 2000 m^2^ grid size.

**Figure 6:**
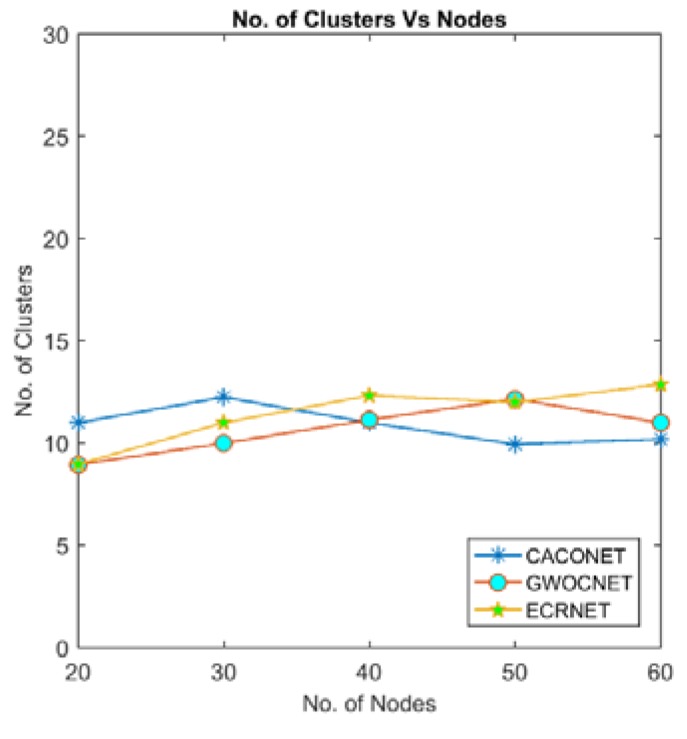
Number of clusters vs. nodes at a 3000 × 3000 m^2^ grid size.

Figure 5 and Figure 6 show the relationship between numbers of clusters and the number of nodes. All three algorithms consider the transmission range to decide the size and number of cluster. Therefore, they have almost same number of the cluster with various degree of nodes and grid size.

### 4.2. Cluster Building Time

This is the time taken by an algorithm to perform clustering. A clustering algorithm takes nodes and their corresponding fitness value as input, and elects CH and their associate members as output. The time elapsed between taking input and producing output is termed the cluster building time. This time denotes the computational complexity of clustering algorithms.

**Figure 7:**
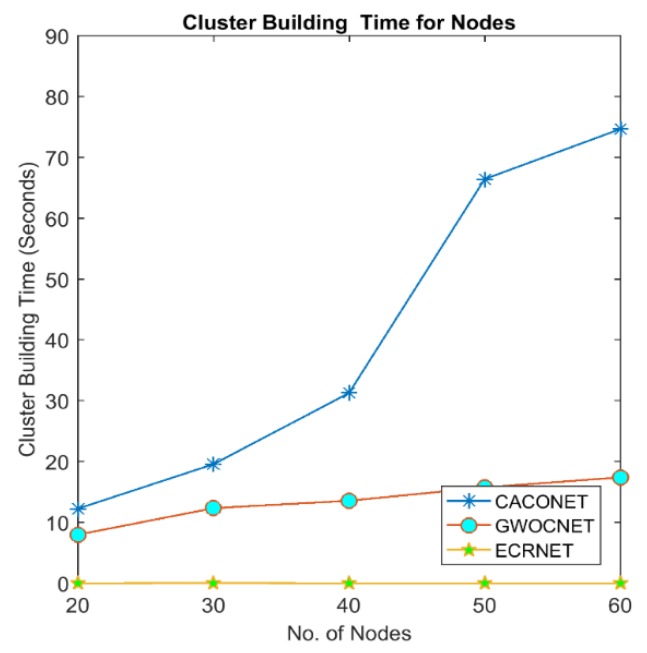
Cluster building time at a 2000 × 2000 m^2^ grid size.

**Figure 8:**
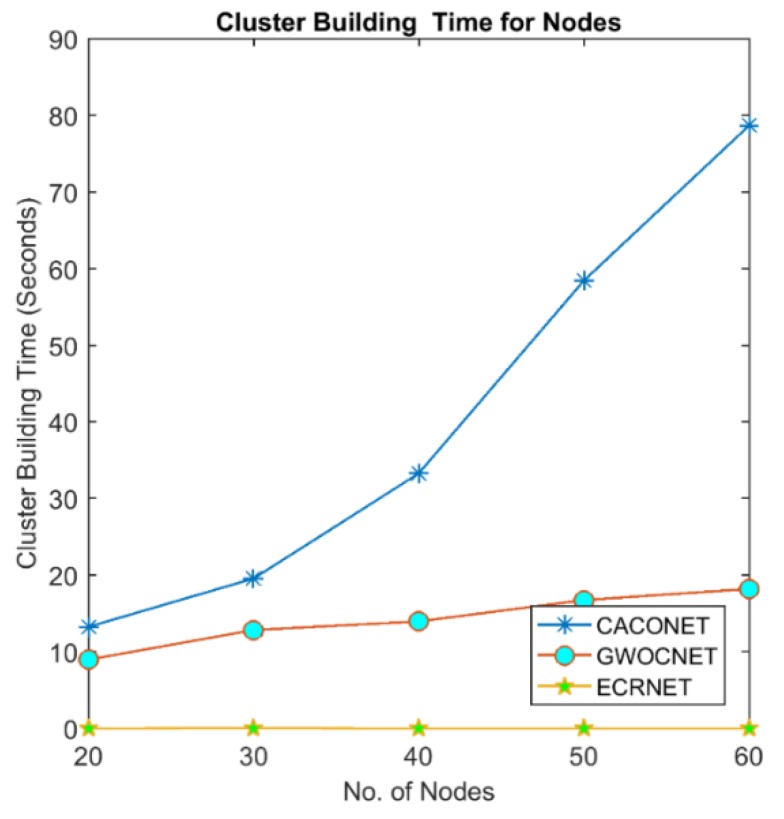
Cluster building time at a 3000 × 3000 m^2^ grid size.

UAV nodes have very low computational power and memory. High cluster building time adversely affects task-oriented performance (sensing and aggregating environmental data). It also consumes more energy and reduces UAVs’ lifetime. Figure 7 and Figure 8 shows that increase in the number of nodes will also increase the cluster building time but EALC is much better than ACO and GWO. The reason behind is that ACO and GWO start with multiple random solutions and iteratively converge towards the optimal solution, whereas, EALC generates only one solution and only adds those nodes that have the highest fitness into a solution. This short cluster building time reduces the delay to find a route. It also saves the nodes energy that is spent during complex computations.

### 4.3. Cluster Lifetime

This is the time elapsed since the formation of a cluster to its destruction. When a clustering algorithm complete execution, the fittest node adopts the role of CH and takes over the responsibility of managing the cluster. The fitness value of CHs decreases with the passage of time. If it falls below some threshold, the clustering algorithm will be recalled. The shorter the cluster lifetime, the more number of times clustering will be recalled. This increases the computational and communicational overhead in the network.

**Figure 9:**
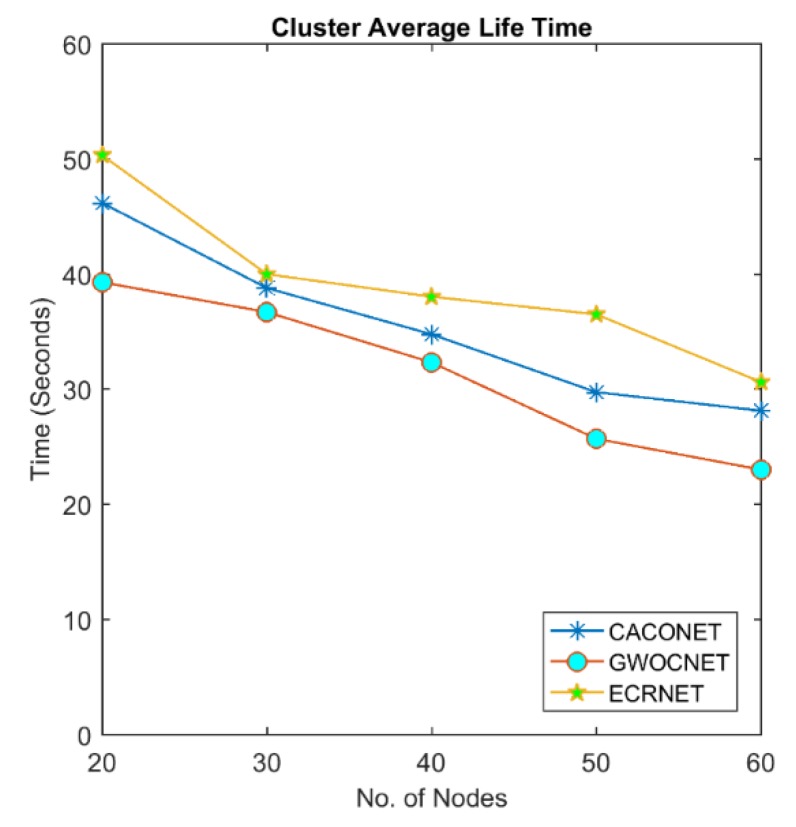
Cluster life time at a 3000 × 3000 m^2^ grid size.

**Figure 10:**
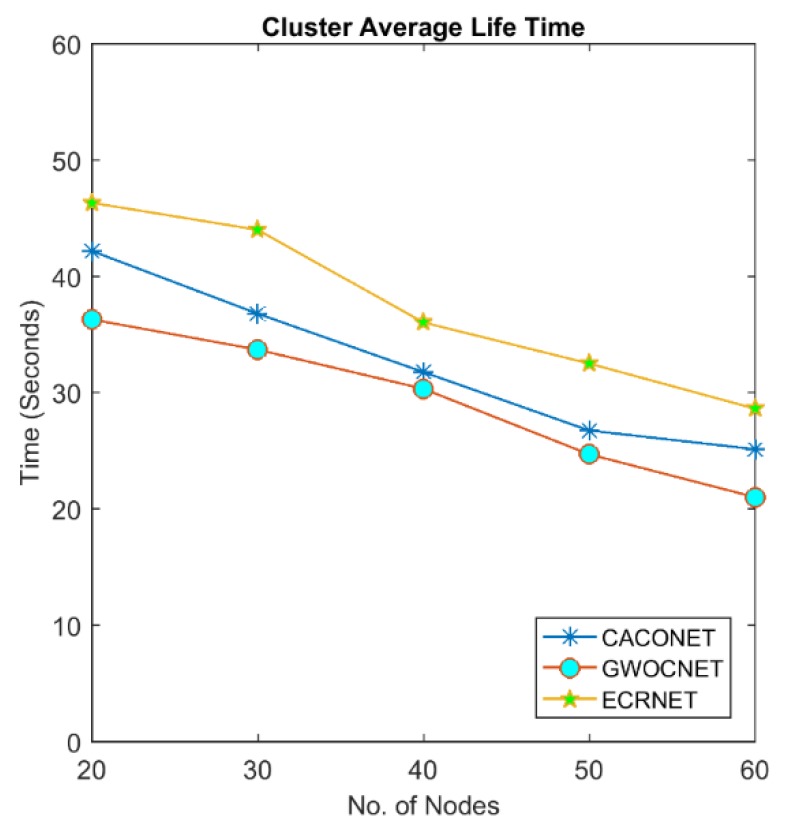
Cluster life time at a 2000 × 2000 m^2^ grid size.

Figure 9 and Figure 10 shows the comparison of cluster lifetime between EALC, CACONET, and GWOCNET. EALC completely outperforms the GWO, whereas it tightly competes with CACONET. The figures also indicate that the cluster lifetime decreases with the increase in the number of nodes in the network. This reduction is due to the fact that cluster has more number of members which cause more changes in the topological arrangement of the network.

### 4.4. Energy Consumption

Energy is one of the scarcest resources in UAVs. Micro UAVs are equipped with a small dry-cell battery which can allow flight operation for only a few minutes; approximately 25 to 30 min. This limited energy puts many hindrances on the wide application of these UAVs. It is highly desirable to optimize the energy in order to enhance the lifetime of a UAV. In UAVs, energy is dissipated by three mechanisms: the energy required to actuate the motor control to fly UAV in the air, energy consumed by different sensors and energy required for communication between UAVs. Communication is a major cause of energy consumption in UAVs. Energy consumed in communication is the sum of energy consumed during transmission and reception [28]:

(8) $$E\;=\;E_{comm}+\;E_{motor}+\;E_{sensor}$$

$$E_{comm}=\;E_{Tx}+E_{Rx}\;$$

where:

(9) $$E_{Tx}=E_{elect}*L+E_{amp}*L*d^{2}$$

and:

(10) $$E_{Rx}\;=\;E_{elect}*L$$

$E_{elect}$ is energy dissipation while running transmitter and receiver circuitry. $E_{amp}$ represents the energy for transmit amplifier. “*L*” shows the number of bits transmitted and “*d*” is the distance between transmitting and receiving nodes. We compute the cumulative energy consumption of whole network using EALC, GWOCNET and CACONET for the period of 120 s.

**Figure 11:**
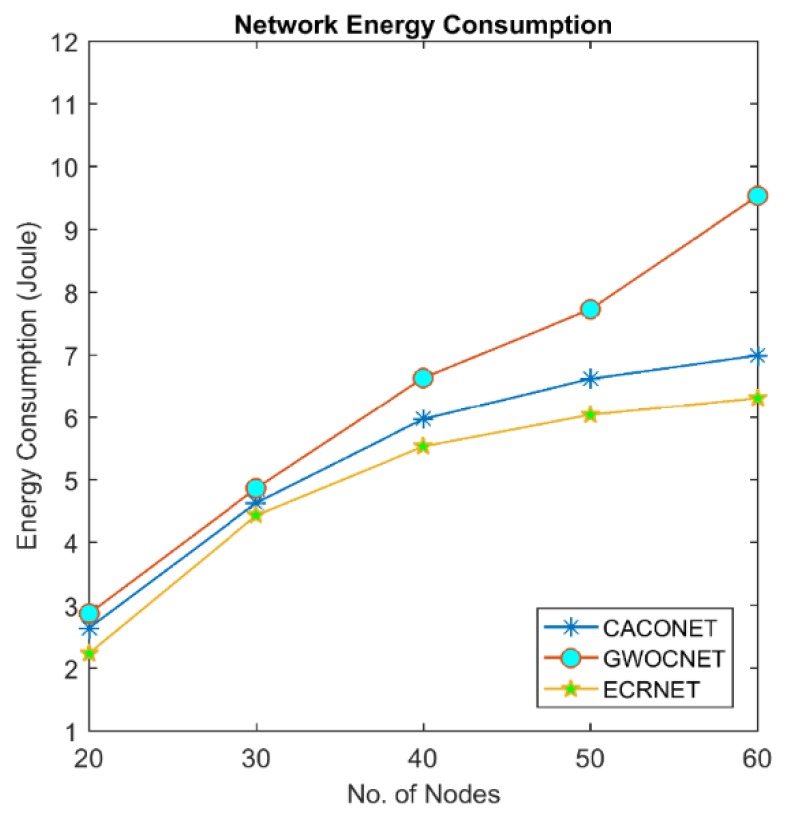
Network energy consumption at 2000 × 2000 m^2^ grid size.

**Figure 12:**
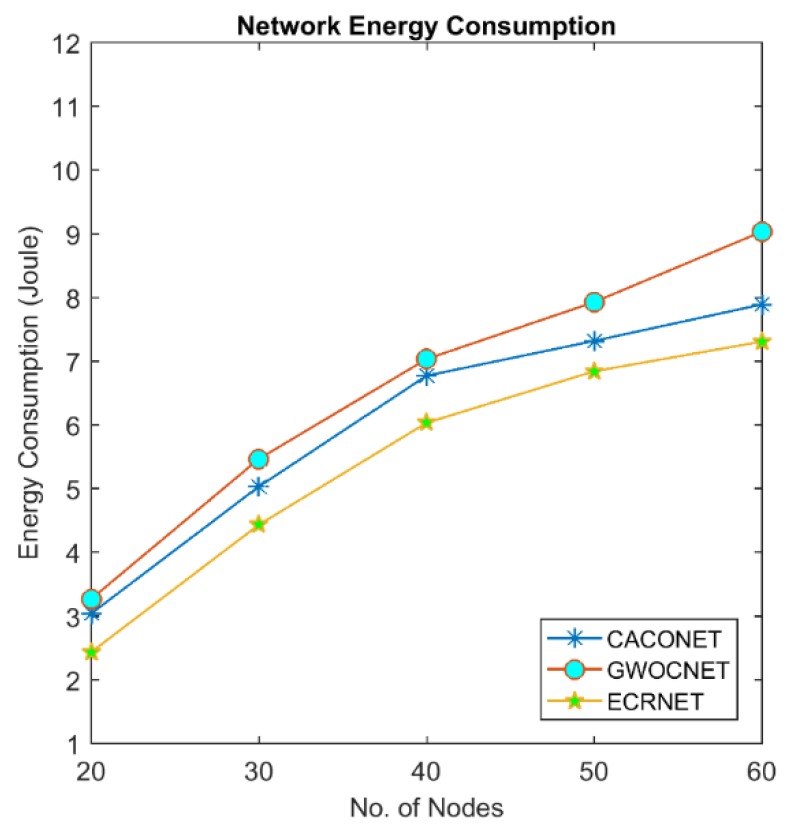
Network energy consumption at 3000 × 3000 m^2^ grid size.

Figure 11 and Figure 12 show that network energy consumption increases with number of nodes. It is clear from the figures that EALC outperforms the other two techniques. Lower energy consumption of EALC is due to the setting of optimal transmission range and election of optimal CHs.

## 5. Conclusions

In this paper, we proposed the EALC model. Two main limitations of fast moving nodes are limited energy and efficient routing. We optimize the routing and save UAVs’ energy by means of controlling their transmission range and efficiently clustering the network. The results of the proposed model are compared with two artificial intelligence algorithms—ACO and GWO—that have also been used for clustering. Results show that EALC competes with the other two approaches in term of number of clusters and cluster lifetime, whereas EALC outperforms them in cluster building time and energy consumption. In future, we will take into account the very high mobility of nodes to perform efficient routing.

## Figures and Tables

**Table 1:** Simulation Parameters.

Parameters	Values
Grid Size	2 × 2 km^2^ and 3 × 3 km^2^
Number of Nodes	20, 30, 40, 50 and 60
Minimum Distance Between Nodes	2 m
Mobility Model	Reference Point Mobility Model
Simulation Runs	10
Simulation Time	120 s
Position Exchange Interval	2 s
Node Energy Level at Start Time	80 Watt Hour
Transmission Range	Dynamic
Transmission Frequency	2.45 GHz
Constant Bit Rate	100 kbps
Receiver Sensitivity	−90 dBm
Stall Iterations (for CACONET and GWOCNETs)	10
